# One year of COVID-19 pandemic on patients with eating disorders, healthy sisters, and community women: evidence of psychological vulnerabilities

**DOI:** 10.1007/s40519-022-01477-5

**Published:** 2022-09-20

**Authors:** Paolo Meneguzzo, Alessandra Sala, Laura Merlino, Enrico Ceccato, Paolo Santonastaso

**Affiliations:** 1 Department of Neuroscience, University of Padova, via Giustiniani 2, 35128 Padua, Italy; 2 Vicenza Eating Disorders Center, Mental Health Department, Azienda ULSS8 “Berica”, Vicenza, Italy; 3 Padova Neuroscience Center, University of Padova, Padua, Italy

**Keywords:** Eating disorders, COVID-19, Posttraumatic, Pandemic, Interpersonal sensitivity, Siblings, Depression

## Abstract

**Purpose:**

The COVID-19 pandemic has been a psychological burden worldwide, especially for individuals with eating disorders (EDs). In addition, the healthy sisters of patients with EDs are known to present specific psychological vulnerabilities. This study evaluates differences between the general population, patients with EDs, and their healthy sisters.

**Method:**

A group of 233 participants (91 patients with EDs, 57 of their healthy sisters and 85 community women) was enrolled in an online survey on general and specific psychopathology 1 year after the beginning of the COVID-19 pandemic. The survey examined associations between posttraumatic symptoms and depression, anxiety, obsessive–compulsiveness, interpersonal sensitivity, and eating-related concerns.

**Results:**

Clinically relevant scores for posttraumatic disorders were found in patients with EDs. Healthy sisters scored similarly to patients for avoidance. Regression analysis showed specific associations between interpersonal sensitivity and posttraumatic symptomatology in patients and healthy sisters, but not in community women.

**Conclusion:**

The psychological burden in patients with EDs is clinically relevant and linked to interpersonal sensitivity, obsessive–compulsiveness, and global symptom severity. Differences between patients, healthy sisters, and community women are discussed regarding vulnerability factors for EDs.

**Level of evidence:**

Level III: evidence obtained from well-designed cohort or case–control analytic studies.

## Introduction

The increased requests for mental health support and interventions linked to eating psychopathology during the COVID-19 pandemic have overwhelmed eating disorder (ED) treatment pathways [1], highlighting the needs for timely recognition and treatment [2] as well as evidencing personal and environmental elements that might play a role in psychopathology.

The COVID-19 pandemic exposed people to traumatic situations due to the disruption of everyday routines, with an exacerbation of specific and general psychopathology [3]; this was defined by some authors as a social or collective trauma [4, 5]. Both patients and the general population reported escalations of eating symptomatology linked to COVID-19 restrictions [6] or exposure to traumatic and uncertain times [7, 8], showing difficulties in managing social isolation, depression, and confinement [9] and portraying symptomatology that is referable at a posttraumatic framework [5]. Longitudinal studies have shown the specific role of COVID-19 in regards to EDs, with patients reporting greater anxiety levels and increased concerns about the impact of the pandemic [10]. Fortunately, protective factors have also been identified, such as family relationships, social support, and therapeutic engagement [11]. Healthcare systems have been overwhelmed by the pandemic’s effects on staff members and the increased support required by patients, showing the psychological burden of the exacerbation of ED symptomatology caused by the loss of in-person support, the feeling of being undeserving of help, and the limitations of online treatment options [12]. Moreover, evaluation of the changes in the effectiveness of treatment before and during the COVID-19 pandemic indicates a reduction in the improvement in patients, calling for evaluations on the possible necessity of specific strategies and procedures to help them [13]. Overall, this evidence showed the possible role of environmental adversity in developing psychological distress, understood as isolation, low mood, anxiety, lack of structure, disruption to routines, and media/social media messages around weight and exercise [14, 15]. However, the data are still preliminary, and new insights are needed from population with specific ED vulnerabilities, to better understand the negative effects that have been recorded [16].

Personal history of maltreatment or traumatic events have been suggested as specific causes for the development and maintenance of EDs [17]. Furthermore, interactions between internal and external elements can modify patients’ physiological responses to subsequent stressful events [18, 19]. Even if no gender differences have been reported in the effects of traumatic events in EDs patients, the literature has shown that women and men have managed the events that have characterized the COVID-19 pandemic—such as confinements—differently due to the internalization of different psychological ideals [20]. The healthy sisters of patients with EDs shared their sisters’ difficulties in terms of emotional regulation, social threats, and executive functions [21, 22]; these elements could increase their hardships in dealing with the changes caused by the pandemic. Moreover, considering that ED patients and their relatives share psychological traits such as perfectionism, anxiety, and specific psychopathology [23], an evaluation of the differences between these particular populations could help determine the specific effects of shared traumatic events.

Despite the elevated psychological risk profiles of ED patients’ sisters, and the evidence of an increase of psychological distress in parents during the pandemic [16], to our knowledge, no study has considered this specific population in the evaluation of the effects of COVID-19; instead, they have focused solely on parents or caregivers [16, 24]. However, literature has shown that comparing ED patients and their sisters enable the evaluation of intra- and interpersonal moderating factors for the impacts of EDs [23]. Therefore, this study aims to evaluate the presence of specific posttraumatic psychological differences due to the COVID-19 pandemic in a sample of patients with EDs, their healthy sisters, as well as a group of unrelated community women, searching for specific features that may explain the different negative effects of the pandemic in each subgroup. Our hypothesis is that specific posttraumatic differences and related psychological elements can be elicited between patients, their sisters, and community peers, contributes to the literature of the field and shows possible specific vulnerabilities that might be considered in future studies. These aspects may help the improvement of treatment approaches and the ED healthcare organizations, which have been overwhelmed by the worsening of patients’ symptoms during the pandemic.

## Methods

This cross-sectional study was performed at the Vicenza Hospital ED Unit, composed of a day hospital and an outpatient service. The study design was approved by the local Ethical Committee and complied with the Declaration of Helsinki. All the participants signed informed consent forms, and the survey was performed via Survey Monkey.

### Sample

Participants were voluntarily enrolled through an online survey about their COVID-19 experiences. Three different samples—patients with ED, healthy sisters (HS) of enrolled patients, and community women (CW)—were recruited between January 2021 and June 2021, one year after the emergence of the COVID-19 pandemic. The inclusion criteria for all participants were as follows: they identify as women; be between 14 and 40 years old, which is the usual range of ages of patients treated at the ED unit; and have no history of psychotic symptoms or severe medical conditions. Patients with EDs fulfilled the DSM-5 criteria for EDs as evaluated in person by a trained psychiatrist. The HS and CW were screened for the exclusion criteria of a personal history of any ED or psychiatric condition with specific items in the online survey; CW were also excluded if they had first-degree relatives with ED diagnoses. Patients were recruited via direct invitations; if present, their sisters were also contacted the same way, while CW were recruited via public announcements on social media.

Different strategies characterized the recruitment process. According to the inclusion and exclusion criteria, all patients treated at the day hospital or the outpatient services were directly enrolled for the study. If present, a trained researcher contacted the closest sister for each patient, screening the inclusion/exclusion criteria and obtaining the consent to participate. For the CW group, participants were enrolled via a public post on local Facebook pages about the search for volunteer participants for a study on the effects of the COVID-19 pandemic on the population; the post was shared on different Facebook pages about community activities. All the participants were volunteers, and none knew the study’s aims.

### Measures

The online questionnaire was composed of a sociodemographic section—including age, weight, height, years of education, personal and relatives’ exposure to COVID-19, and the current lockdown condition—and three different validated questionnaires in Italian: the eating disorder examination questionnaire (EDE-Q), the brief symptom checklist (SCL-58), and the revised version of the Impact of Event Scale (IES-R). All participants were specifically instructed to focus on pandemic-related experiences when filling the survey.

For the healthy sisters and CW participants, specific questions were included in the survey to evaluate their psychological/psychiatric history, with a yes/no answer required: “Are you currently being treated for an eating disorder (e.g., anorexia nervosa or bulimia nervosa)?”; “Have you ever been treated for an eating disorder?”; “Are you currently being treated by a psychologist or psychiatrist for a specific your condition (depression, anxiety, bipolar disorder, etc.)?”; and “Have you ever been treated for a psychological problem (depression, anxiety, bipolar disorder, etc.) in the past?”.

The EDE-Q is a well-known validated 28-item self-report questionnaire specifically structured to evaluate eating symptomatology and attitudes [25, 26]. The measure contains four subscales, and higher total scores reflect greater eating-related pathology. Specific items allow the researcher to evaluate the frequency of particular clinical features—binge-eating episodes, self-induced vomiting, laxative and diuretics misuses, use of exercise as compensation—per month. In the current investigation, Cronbach’s *α* was 0.98 for the global score, 0.88 for the restraint subscale, 0.87 for the eating concern subscale, 0.94 for the shape concern subscale, 0.88 for the weight concern subscale.

The SCL-58 is a well-known self-report questionnaire derived from SCL-90R, and it is widely used to evaluate general psychiatric symptoms and psychological distress [27–29]. It provides a total score and five subscales: somatization, obsessive–compulsive, interpersonal sensitivity, depression, and anxiety. Each item is rated on a five-point scale, and higher total scores reflect greater symptomatology. In the current investigation, Cronbach’s *α* was 0.94 for the global severity index, 0.90 for the somatization subscale, 0.88 for the obsessive–compulsive subscale, 0.83 for the interpersonal sensitivity subscale, 0.88 for the depression subscale, and 0.85 for the anxiety subscale.

The IES-R is a 22-item self-report questionnaire that assesses subjective distress caused by specific events such as the COVID-19 pandemic [30, 31]. It is comprised of three different subscales (avoidance, intrusion, and hyperarousal) and a total score. Again, the participants were explicitly instructed to respond to the questionnaire in relation to their experiences of the pandemic. The clinical relevance cut-off for the impact of an event is 24. A score of 33 or above represents a possible diagnosis of posttraumatic stress disorder [32]. In the current investigation, Cronbach’s α was 0.97 for the total score, 0.81 for the avoidance subscale, 0.90 for the intrusion subscale, and 0.84 for the hyperarousal subscale.

### Data analysis

Demographic information and specific and general psychopathology differences between the groups were evaluated using different analysis of variance (ANOVA) tests. In addition, a Bonferroni approach was used in the post-hoc analysis. Lockdown conditions were classified according to the Italian legislation: yellow meant that people were not allowed to leave their homes from 10 pm to 5 am; orange meant that people were allowed to leave their towns only for work, basic needs, or health-related reasons; and red meant that people could not leave their towns except for health or work-related reasons. The distribution of lockdown conditions was evaluated using a Chi-square approach. Due to their equal distribution, comparison between underweight and normal weight patients were performed with a *t* test analysis. Instead, considering the different sample sizes, the patients were compared with the Mann–Whitney test to find differences linked to the various treatments at the evaluation. Furthermore, correlation between clinical features (binge-eating episodes, self-induced vomiting, laxative and diuretics misuses, EDE-Q subscales) and IES-R subscales were evaluated with Pearson’s correlation analysis. For the clinical features were used the specific items in the EDE-Q questionnaires. Hierarchical multiple linear regression analyses were applied to evaluate the associations between general psychopathology and EDE-Q global scores for posttraumatic symptoms linked to COVID-19. The IES-R total score was used as the dependent variable, while the SCL-58 subscales (Step 1) and the EDE-Q total score (Step 2) were used as independent variables. The effect size was evaluated with Cramer’s V for the chi-square and partial *η*^2^ for the ANOVA. All the analyses were performed using IBM-SPSS software vers. 25 with the alpha set at *p* < 0.05.

## Results

A total of 254 women participated in the survey; of these, ten were excluded due to incomplete responses, and 11 were excluded from the HS or CW subgroups due to the presence of a personal or, for the CW subgroup, a first-degree relative’s history of ED. Therefore, the final sample included 233 participants: 91 ED patients, 57 HS, and 85 CW. The ED group consisted of: 48 females with anorexia nervosa, 24 with bulimia nervosa, eight with binge-eating disorder, and 11 with other specified eating disorders. The groups reported a significant difference only in regards to their average body mass index (BMI), which could be linked to the underweight of individuals with anorexia nervosa; ED group: 21.10 ± 6.49 years old, body mass index (BMI) of 19.50 ± 5.47 kg/m^2^, and 12.56 ± 2.77 years of education, all cisgender women; HS group: 21.52 ± 6.91 years old, BMI of 21.27 ± 3.70 kg/m^2^, and 12.77 ± 1.84 years of education, 55 cisgender women and two queer women; CW group: 21.98 ± 2.76 years old, BMI of 21.22 ± 2.39 kg/m^2^, and 13.34 ± 1.92 years of education, 79 cisgender women and 6 queer women. Due to the small sample of queer women, no subsample analysis were performed for genders for absence of statistical power. Regarding exposure to COVID-19, no differences were found across the groups in terms of personal infection (participants with previous infections included 9.30% of ED patients, 3.5% of HS and 10.6% of CW; *χ*^2^ = 3.82, *p* = 0.15), infection in cohabitant relatives (59.3% of ED patients, 60.8% of HS and 76.3% of CW; *χ*^2^ = 1.74, *p* = 0.36), or current lockdown situation (*p* = 0.31). The results of the psychopathological self-report evaluations are reported in Table 1. Differences were found between ED patients, HS, and CW, as the patients showed higher scores than the other groups in both general and specific psychopathology and their quantifications of the effects of the pandemic in their lives. See Table 1 for further details.

**Table 1:** Lockdown condition and psychological description of the participants

	ED		HS		CW		*χ*^2^	*V*
	*n*	%	*n*	%	*n*	%		
Housing								
Alone	2	2	3	5	7	8	9.86	0.13
Original family	79	87	40	70	60	71		
Husband	8	9	8	14	7	8		
Friends	2	2	6	11	11	13		
Lockdown area								
Yellow	57	63	32	56	45	53	4.81	0.31
Orange	26	28	14	25	29	34		
Red	8	9	11	19	11	13		

	*M*	SD	*M*	SD	*M*	SD	F (2, 207)	*η*^2^	Post hoc
EDE-Q									
Restraint	3.06	1.77	0.82	1.31	1.02	1.23	50.87***	0.32	ED > HS*** ED > CW***
Eating concern	3.13	1.50	0.91	1.47	0.91	1.09	62.70***	0.37	ED > HS*** ED > CW***
Shape concern	4.36	1.62	1.86	1.90	2.15	1.55	46.80***	0.30	ED > HS*** ED > CW***
Weight concern	3.56	1.87	1.63	1.62	1.47	1.45	33.82***	0.24	ED > HS*** ED > CW***
Global	3.53	1.48	1.31	1.48	1.39	1.19	58.61***	0.35	ED > HS*** ED > CW***
SCL-58									
SOM	1.63	.92	1.04	.84	0.90	.66	17.31***	0.14	ED > HS*** ED > CW***
OC	1.59	1.06	1.07	.85	0.96	.81	9.22***	0.08	ED > HS* ED > CW***
IS	1.42	.96	0.99	.85	0.91	.79	10.38***	0.09	ED > HS** ED > CW***
D	1.66	.90	1.07	.81	1.07	.81	11.96***	0.10	ED > HS*** ED > CW***
A	1.65	.95	1.03	.77	1.11	1.01	9.08***	0.08	ED > HS*** ED > CW***
GSI	1.65	.90	1.08	.78	1.04	.79	14.86***	0.12	ED > HS*** ED > CW***
IES-R									
Avoidance	11.39	7.00	9.26	5.93	7.50	5.38	8.22***	0.07	ED > CW*** HS > CW*
Intrusion	10.01	7.40	7.10	5.80	8.06	6.45	3.47*	0.03	ED > HS* ED > CW*
Hyperarousal	9.29	5.84	6.96	4.81	7.01	5.44	3.48*	0.03	ED > CW*
IES-R Total	30.69	18.63	23.33	15.35	23.45	15.72	4.88**	0.04	ED > HS* ED > CW**

*ED* patients with an eating disorder, *HS* healthy siblings of a patients, *CW* community women, *SOM* somatization, *OC* obsessive compulsive, *IS* interpersonal sensitivity, *D* depression, *A* anxiety, *GSI* global severity index

^*^p < 0.05, ^**^p < 0.01, ^***^p < 0.001

Using hierarchal multiple linear regression analyses, we evaluated the relationship between psychological features and the posttraumatic effects of the COVID-19 pandemic. As reported in Table 2, we found different results for the groups: ED patients showed a significant regression model for IES-R Global Score with obsessive–compulsive, interpersonal sensitivity, and global severity as predictors; in contrast, HS reported a significant regression model with interpersonal sensitivity and depression as predictor, and the CW group only reported a significant regression model for IES-R Total Score with depression.

**Table 2:** Results of Hierarchical multiple regression analyses of IES-R Total score on predictors

Predictor variables	ED				HS				CW			
	*B*	*β*	95% CI for *B*		*B*	*β*	95% CI for B		*B*	*β*	95% CI for *B*	
			LL	UL			LL	UL			LL	UL
Step 1												
SOM	0.11	0.01	− 7.17	7.39	7.22	0.39	− 7.12	21.57	0.70	2.25	− 3.78	5.18
OC	− 8.72	− 0.47**	− 14.53	− 2.90	− 5.64	− 0.31	− 12.62	1.34	− 2.79	4.82	− 12.39	6.81
IS	11.08	0.61**	4.13	18.02	10.67	0.59**	4.73	16.61	1.18	4.60	− 7.98	10.35
D	6.80	0.32	− 1.27	14.88	4.51	0.24*	1.31	10.33	13.84	3.10**	7.66	20.02
A	− 2.42	− 0.12	− 8.89	4.01	− 2.99	− 0.15	− 10.05	4.08	2.13	2.34	− 2.54	6.81
GSI	8.46	0.41*	1.34	18.27	1.94	0.10	− 16.57	20.46	1.03	7.94	− 14.78	16.85
Step 2												
SOM	− 1.29	− 0.06	− 9.02	6.44	6.94	.38	− 7.82	21.71	0.16	2.18	− 4.19	4.50
OC	− 8.56	− 0.46	− 14.38	2.75	− 5.48	− 0.31	− 12.71	1.75	− 0.76	4.72	− 10.16	8.65
IS	10.78	0.59**	3.82	17.74	10.70	0.59**	4.68	16.71	0.81	4.44	− 8.04	9.66
D	6.48	0.31	− 1.61	14.57	4.54	0.24*	1.36	10.43	13.38	3.00**	7.40	19.36
A	− 2.13	− 0.11	− 8.62	4.36	− 2.88	− 0.15	− 10.10	4.33	1.63	2.27	− 2.90	6.15
GSI	8.60	0.42*	1.20	18.41	1.72	.09	− 17.12	20.57	− 1.10	7.70	− 16.45	14.26
EDE-Q Global	1.25	0.10	− 1.08	3.58	0.21	0.02	− 1.83	2.26	2.49	0.98	− 0.53	4.54

ED: for Step 1 *R*^*2*^ = 0.66, *F* = 25.73, *p* < 0.001; for Step 2 *R*^*2*^ ∆ = 0.005, *p* < .001. HS: for Step 1 *R*^*2*^ = 0.73, *F* = 20.05, *p* < 0.001; for Step 2 *R*^*2*^ ∆ = 0.001, *p* < 0.001. CW: for Step 1 *R*^*2*^ = 0.58, *F* = 15.687, *p* < .001; for Step 2 *R*^*2*^ ∆ = 0.032, *p* < 0.001

*CI* confidence interval, *LL* lower limit, *UL* upper limit, *ED* patients with an eating disorder, *HS* healthy siblings of a patients, *CW* community women, *SOM* somatization, *OC* obsessive compulsive, *IS* interpersonal sensitivity, *D* depression, *A* anxiety, *GSI* global severity index

^*^p < 0.05, ^**^p < 0.01

### Evaluation of clinical characteristics interactions

Examining the role of a condition of underweight in patients, no differences were found between the 48 underweight and 43 normal weight patients for general psychopathology or posttraumatic symptoms. The only difference that emerged was the mean scores of the restraint subscale of the EDE-Q questionnaire, which is linked to the different clinical presentation: underweight 3.39 ± 1.69, normal weight 2.63 ± 1.67, *t* (89) = 2.16, *p* = 0.03.

Correlation analyses between clinical features and posttraumatic symptomatology related to COVID-19 pandemic have reported specific significance relationships that are presented in Table 3.

**Table 3:** Correlation analyses between clinical features and IES-R scores in ED patients

	Avoidance	Intrusion	Hyperarousal	IES-R total
Restraint	*r* = 0.307 *p* = 0.003	*r* = 0.512 *p* < 0.001	*r* = 0.354 *p* = 0.001	*r* = 0.434 *p* < 0.001
Eating concern	*r* = 0.476 *p* < 0.001	*r* = 0.493 *p* < 0.001	*r* =0 .490 *p* < 0.001	*r* = 0.534 *p* < 0.001
Shape concern	*r* = 0.353 *p* = 0.001	*r* = 0.393 *p* < 0.001	*r* = 0.405 *p* < 0.001	*r* = 0.420 *p* < 0.001
Weight concern	*r* = 0.336 *p* = 0.001	*r* = 0.396 *p* < 0.001	*r* = 0.433 *p* < 0.001	*r* = 0.424 *p* < 0.001
Global	*r* = 0.414 *p* = 0.001	*r* = 0.508 *p* < 0.001	*r* = 0.476 *p* < 0.001	*r* = 0.512 *p* < 0.001
Binge-eating episodes	*r* = − 0.001 *p* = 0.991	*r* = − 0.045 *p* = 0.673	*r* = 0.098 *p* = 0.355	*r* = 0.012 *p* < 0.001
Self-induced vomiting	*r *= 0.096 *p* = 0.365	*r* = 0.042 *p* = 0.691	*r* = 0.112 *p* = 0.289	*r* = 0.089 *p* < 0.001
Laxative and diuretics misuses	*r* = 0.313 *p* = 0.003	*r* = 0.337 *p* = 0.001	*r* = 0.336 *p* = .001	*r* = 0.361 *p* < 0.001
Use of exercise	*r* = 0.169 *p* = 0.109	*r* = 0.288 *p* = 0.006	*r* = 0.248 *p* = 0.018	*r* = 0.258 *p* = 0.014

In the table are reported the values of the Pearsons’r, with the *p*values

### Inpatients vs. outpatients

Patients recruited for the study were from both the day hospital (17 out of 91) and the outpatient services. When comparing these groups, some demographic differences linked to the nature of the day hospital program emerged, as it is more focused on severe restrictive behaviors. Examining the psychopathology scores, differences were found only for eating psychopathology, with day hospital patients reporting higher scores due to their greater clinical severity. See Table 4.

**Table 4:** Differences between patients in the Day Hospital program and the outpatient service

	Outpatients		DH patients		*Z*(91)	*p*
	*M*	*SD*	*M*	*SD*		
Age	21.21	6.65	19.98	6.16	− 0.34	0.062
BMI	20.53	5.38	16.65	4.86	− 4.29	< 0.001
EDE-Q						
Restraint	2.78	1.76	4.21	1.39	− 2.91	0.002
Eating concern	3.02	1.58	3.62	1.11	− 1.34	0.085
Shape concern	4.14	1.68	5.19	0.92	− 2.26	0.013
Weight concern	3.30	1.94	4.51	1.22	− 2.19	0.009
Global	3.31	1.52	4.38	0.99	− 2.58	0.003
SCL-58						
SOM	1.53	0.88	1.93	1.02	− 1.44	0.150
OC	1.52	1.01	1.82	1.18	− 0.93	0.278
IS	1.33	0.89	1.75	1.13	− 1.30	0.361
D	1.59	0.87	1.88	0.98	− 1.16	0.259
A	1.59	0.95	1.83	0.88	− 1.13	0.253
GSI	1.58	0.86	1.89	0.98	− 1.12	0.441
IES-R						
Avoidance	1.36	0.87	1.31	0.93	− 0.239	0.828
Intrusion	1.13	0.94	1.44	1.05	− 1.16	0.234
Hyperarousal	1.53	0.93	1.70	1.10	− 0.412	0.483
IES-R total	1.32	0.84	1.46	0.96	− 0.436	0.560

*SOM* somatization, *OC* obsessive compulsive, *IS* interpersonal sensitivity, *D* depression, *A* anxiety, *GSI* global severity index

## Discussion

The present study explored the psychological effects of a year of exposure to the COVID-19 pandemic in patients with EDs and their HS by comparing them to a group of CW. Our main hypothesis was the presence of different posttraumatic responses in individuals with EDs, their HS, and a sample of CW. Our data showed that participants with EDs reported higher scores in all the subscales, indicating the presence of more specific posttraumatic responses to distressing experiences like the COVID-19 pandemic [20], and corroborating the evidence of the relationships between traumatic events and general and ED psychopathology [33].

As expected, no differences emerged between the HS and CW groups regarding specific and general symptomatology. However, HS reported higher scores than CW for avoidance with no differences between HS and ED patients. Interestingly, avoidance has been reported to be a core construct in posttraumatic symptomatology in EDs, implicating its role in the activation of ED and trauma-related symptomatology [34]. While the existing literature has already suggested the presence of posttraumatic effects of the COVID-19 pandemic in patients with EDs [35], our data provide a specific profile for their healthy sisters that could encourage further study about the effects of environmental adversities in subjects with EDs and its possible effects on genotype [36]. This interaction might not only be characterized by the presence of different psychological and temperamental traits [23, 36] but also by the presence of neurobiological differences that might need to be evaluated [37]. Our data show that traumatic events might produce similar posttraumatic symptomatology in the healthy sisters of patients with EDs, even if they do not reach a clinically significant level of posttraumatic disorder. Biological and environmental interactions are still an open field of research in EDs, but a growing body of data highlights the role of these factors in the differences in life trajectories of sisters with and without EDs [38]. Indeed, several models have been proposed to explain the connections between traumatic or adverse events and EDs [39], and a wealth of data corroborates the presence of a blunted stress response in distressed patients with EDs [37, 40]. From this perspective, COVID-19 may be considered a psycho-social stressor with possible cognitive and emotional effects in ED patients [30, 41]. Moreover, another noteworthy result was the presence of an association between posttraumatic symptoms and interpersonal sensitivity in both patients and their sisters. This association underlined the possible role of insufficient social support or social abilities in the face of complex events [35]; these factors have already been cited as core elements in the development and maintenance of eating psychopathology [42–44], and should be explored in more detail in patients’ sisters in future studies. Laboratory tasks have explored and reported specific negative effects of social interaction in individuals with EDs, indicating the presence of interpretation bias, fear for negative evaluations, and less physiological arousal in social interactions [43, 45–47]. Conversely, depressive symptoms have been confirmed as related to posttraumatic symptomatology in community samples during the COVID-19 pandemic [48], with differences in patients which could be explained by the presence of higher scores in women with EDs for both depressive and posttraumatic symptomatology. Finally, only ED patients showed an association between posttraumatic symptoms and obsessive-compulsiveness; however, this is in line with previous literature about the pandemic’s role in exacerbating general and ED symptomatology in ED patients [6].

Looking at clinical features, several connections have been found between posttraumatic symptoms and binge or compensatory behaviors in patients with ED. These correlations are consistent with the existing literature about trauma in EDs, the hypothesis being that they might be a mental escape from negative affects [49]. Interestingly, these data have been reported by different population studies as effects of the COVID-19 pandemic, even in the general population [50, 51]. Moreover, while these elements have already been classified as fundamental by trauma-informed treatments, there is still work to be done in their integration into evidence-based treatments [52]. These data corroborate the implementation of specific interventions focused on the development of effective and adaptive strategies to cope with life’s adversities and engaging with valued activities to help those struggling with EDs to survive in years to come.

### Strength and limits

The cross-sectional and self-reported nature of the questionnaires limits the reliability of the results, even if the differences between the groups are robust. However, while the exclusion of sisters with present or past histories of psychiatric conditions might improve the statistical strength of our results by clearing the samples of confounders, it might also be considered a limit for the generalizability of the study. In addition, the generalizability is further restricted by the exclusion of men. Other information, such as the participants’ occupations and the duration of their EDs, is missing from this study and should be considered in the future. Moreover, future studies should separate the first national lockdown of the spring of 2020 from the following partial or total lockdown conditions.

## Conclusion

In conclusion, this study shows the presence of a psychological impairment clinically defined as posttraumatic symptomatology in patients with EDs after one year of containment measures to combat the COVID-19 pandemic. Additionally, it also indicates the shared role of interpersonal sensitivity in ED patients and their healthy sisters in the development of posttraumatic psychological responses linked to stressful events, as well as the specific role of obsessive–compulsive and depressive symptoms in individuals with and without EDs. These results corroborate evidence of the role of the interaction between biological substrates and non-environmental features linked to adversity in the development and maintenance of EDs. However, more evidence—including data from men—is needed for more robust generalizability.

## What is already known on this subject?

The COVID-19 pandemic has exacerbated ED psychopathology in the general population with an increase in the request for specific treatments. Patients with an ED presented a specific profile associated with traumatic events that might be considered a vulnerability aspect of this increase in prevalence.

## What this study adds?

This study evaluates the effects of exposure to the constraints of the COVID-19 pandemic 1 year after its beginning in a sample of women (ED patients, their healthy sisters, and community women), examining psychological features that might play a role in treatment approaches and in the comprehension of the pandemic’s psychological effects. Our data show the possible role of interpersonal sensitivity in the psychological burden of patients and families; in addition, it indicates that depression plays a role in the posttraumatic symptomatology of community women, providing a possible focus of investigation.

## Data Availability

The datasets used and analyzed during the current study are available from the corresponding author on reasonable request.
